# Hesperetin improves cognitive impairment in scopolamine-induced C57BL/6 mice

**DOI:** 10.29219/fnr.v70.14414

**Published:** 2026-10-27

**Authors:** Ha-Rin Moon, Jung-Mi Yun

**Affiliations:** 1 Department of Food and Nutrition, Chonnam National University, Gwangju, South Korea; 2 Global Convergence Research Division, World Institute of Kimchi, Gwangju, South Korea

**Keywords:** cognitive impairment, hesperetin, scopolamine, inflammation, apoptosis

## Abstract

**Background:**

Cognitive impairment limits daily functioning and is closely linked to neuroinflammation and neuronal death caused by oxidative stress. Hesperetin, a bioactive flavonoid aglycone from citrus peels, remains relatively unexplored despite its therapeutic potential.

**Objective:**

This study evaluated hesperetin in scopolamine (SCO)-induced cognitive impairment in C57BL/6 mice.

**Design:**

Memory impairment was induced using SCO (1 mg/kg). Mice were divided into control (*n* = 10), SCO (*n* = 10), and SCO with hesperetin (25 or 50 mg/kg/day, *n* = 10) groups. Treatments were administered daily for 28 days. Spatial memory performance was assessed using the Y-maze test. Brain histology was examined through hematoxylin and eosin staining. Malondialdehyde (MDA) levels were measured to evaluate lipid peroxidation, and acetylcholinesterase (AChE) activity was analyzed. The underlying mechanism was investigated using Western blotting and quantitative polymerase chain reaction, focusing on the AKT (protein kinase B)/CREB (cAMP-response element binding protein)/BDNF (brain-derived neurotrophic factor) signaling pathway.

**Results:**

Hesperetin significantly improved cognition, increasing spontaneous alternation in the Y-maze (*P* < 0.05). It also mitigated histopathological changes, such as neuronal contraction and morphological abnormalities, and improved lipid profiles. Hesperetin reduced AChE activity, MDA levels, and the expression of proinflammatory markers (tumor necrosis factor-α, cyclooxygenase-2, and nuclear factor-κB). The AKT/CREB/BDNF pathway, critical for long-term memory formation, was activated by hesperetin through increased phosphorylation of AKT and CREB and elevated BDNF expression. Hesperetin enhanced H3K9 and H3K27 acetylation, reduced H3K4 and H3K27 dimethylation, and restored sirtuin 1–histone deacetylase balance, all of which are associated with memory formation.

**Conclusion:**

Overall, hesperetin improved cognition in SCO-treated mice, highlighting its potential as a therapeutic candidate for neurodegenerative disorders.

## Popular scientific summary

- Hesperetin significantly improved behavioral deficits in a scopolamine-induced mouse model of cognitive impairment, attenuated brain tissue alterations such as neuronal shrinkage, and reduced MDA and AChE levels.
- Hesperetin suppressed apoptosis by modulating the Bax/Bcl-2 ratio and inhibited inflammation by downregulating the expression of proinflammatory genes, including TNF-α, IL-6, and other NF-κB target genes in scopolamine-induced mice.
- Hesperetin mitigated cognitive impairment associated with neurodegenerative diseases by enhancing the AKT/CREB/BDNF signaling pathway and regulating histone acetylation and methylation in the scopolamine-induced cognitive impairment mouse model.

The global increase in the elderly population has led to a rising prevalence of neurodegenerative diseases, chronic conditions characterized by progressive brain degeneration (1). Cognitive impairment arises from multiple factors, including aging, acetylcholine (ACh) depletion, drug addiction, inflammation, brain injury, or neurological disorders. This decline is linked to complex mechanisms, particularly immunological and cholinergic dysfunction in the brain. The cholinergic system plays a crucial role in memory, learning, dendritic growth, and neuronal development and differentiation (2). In neurodegenerative conditions, cholinesterase enzymes such as acetylcholinesterase (AChE) and butyrylcholinesterase are highly active in the hippocampus, accelerating ACh breakdown into choline and acetyl-CoA, contributing to cholinergic dysfunction (3). Cholinesterase inhibitors are the most widely used pharmacological treatment to address neurotransmitter imbalances in the brain (4). However, their use is limited by side effects such as gastrointestinal discomfort and insomnia (3). Scopolamine (SCO), a muscarinic cholinergic receptor antagonist, impairs learning and memory by ACh–receptor interactions and disrupting synaptic transmission (5). This interference compromises synaptic plasticity and damages synaptic structure and function, which are critical for learning and memory consolidation (6). SCO also increases AChE activity, reduces cAMP-response element binding protein (CREB) and brain-derived neurotrophic factor (BDNF) levels, and elevates the expression of inflammatory mediators (7). In animal models, it reduces *CREB* binding and *BDNF* expression, thereby impairing memory formation and synaptic plasticity (8). Studies have confirmed that SCO disrupts cholinergic neurotransmission and impairs cognitive performance (7). Although SCO is used therapeutically to treat nausea and depression, it is also associated with memory deficits and disorientation (9).

SCO-induced oxidative stress accelerates cognitive dysfunction by promoting inflammation and apoptotic signaling in brain cells (10). It activates inflammatory pathways by upregulating nuclear factor-κB (NF-κB) and proinflammatory cytokines, which are predominantly overexpressed in the brain in regions affected by neuroinflammation (11). This inflammatory response contributes to oxidative stress and mitochondrial dysfunction, ultimately inducing neuronal apoptosis through Bax activation and Bcl-2 downregulation (12). Neuronal death in the central nervous system is associated with cognitive impairment, with oxidative stress recognized as a significant contributor to SCO-induced cognitive deficits in both cellular and animal models (13). Moreover, oxidative stress correlates with neuronal loss, a central mechanism in the pathogenesis of neurodegenerative diseases (7). Consistent with these findings, postmortem studies frequently reveal neuronal cell death in patients with neurodegenerative disorders (14). In addition to oxidative stress and neuroinflammation, dysregulated lipid metabolism has recently been recognized as an important contributor to cognitive decline and neurodegenerative diseases (15). Altered lipid homeostasis has been associated with oxidative stress, neuroinflammation, cerebrovascular dysfunction, and impaired neuronal function, all of which contribute to cognitive impairment (16).

SCO administration in animals reduces CREB binding and BDNF levels, impairing memory formation and synaptic plasticity (8). The Tropomyosin receptor kinase B TrkB/CREB/BDNF signaling pathway plays a pivotal role in promoting neurogenesis (17). CREB activation enhances hippocampal cell proliferation, neurogenesis, differentiation, and survival by promoting *BDNF* transcription (18). BDNF, a crucial neurotrophin, supports synaptic development, plasticity, and neuronal survival; its reduction is associated with structural deterioration and progressive neuronal atrophy in Alzheimer’s disease (AD) (18). Recent studies suggest that targeting the BDNF/CREB/TrkB pathway may provide neuroprotection in cellular and animal models of neurodegenerative disease (8).

Epigenetic regulatory mechanisms, including chromatin remodeling, DNA methylation, and histone modifications, are essential for neural development and function. Aberrant histone methylation and acetylation have been implicated in the arrest of neural stem cell proliferation, microglial dysfunction, inflammatory processes, memory impairment, cognitive decline, and neurodegenerative diseases such as schizophrenia (19). Disruption of these processes may contribute to the onset and progression of neurodegenerative diseases (19). Recent studies have highlighted altered histone acetylation and methylation patterns associated with cognitive decline in mouse models (19). In particular, the genes Sirtuin 1 (*SIRT1*) and histone deacetylases (HDACs), specifically *HDAC2* and *HDAC4*, are strongly associated with the development of neurodegenerative diseases (20). SIRT1, a NAD+-dependent deacetylase in the sirtuin family, plays a crucial role in regulating neuroinflammation and suppressing the NF-κB pathway, thereby reducing inflammatory responses (21). Similarly, inhibition of HDAC2 and HDAC4 has demonstrated potential in alleviating neuroinflammation in animal models (22). Ongoing research is investigating natural compounds with minimal side effects, such as gastrointestinal disturbances and insomnia, for the prevention and treatment of dementia. Plant-derived compounds, including flavonoids, tannins, alkaloids, and terpenes, have demonstrated neuroprotective properties through antioxidant, anti-inflammatory, anti-amyloid, and AChE-inhibitory mechanisms (23). Notably, sulforaphane enhances hippocampal synaptic activity by upregulating *BDNF* and *CREB* expression in SCO-treated mice, thereby improving memory function (18). Hesperetin, a bioflavonoid first isolated from citrus peels, exhibits various physiological effects, including anticancer and cardiovascular benefits (24). However, its potential for improving cognition in memory impairment models remains unexplored. This study investigated the memory-enhancing effects and underlying mechanisms of hesperetin in an SCO-induced cognitive impairment mouse model.

## Materials and methods

### Animals and diet

Male C57BL/6N mice (6 weeks old) were obtained from Orient Bio Co. (Gyeonggi-do, South Korea). The animals were housed under controlled environmental conditions, with temperatures maintained between 20 and 25°C and humidity levels at 55–60%. A 12-h light–dark cycle was maintained, and mice had unrestricted access to food and water. To standardize nutritional status prior to dietary experiments, all mice were fed the AIN-93G diet (Saeronbio Inc., Gyeonggi-do, South Korea) for 1 week. Subsequently, the mice were randomly assigned to four experimental groups (*n* = 10 per group): a normal control group (CON), an SCO-treated group (SCO; 1 mg/kg/day), and two hesperetin-treated groups receiving either 25 mg/kg/day or 50 mg/kg/day of hesperetin in combination with SCO. The doses of hesperetin (25 and 50 mg/kg/day) were selected based on previous in vivo studies demonstrating antioxidant and neuroprotective effects without apparent toxicity (25–28). After a 1-week acclimation period, hesperetin was orally administered once daily at doses of 25 or 50 mg/kg/day for 4 weeks, starting at 7 weeks of age. To establish the SCO-induced cognitive impairment model, SCO (1 mg/kg/day) was administered by daily intraperitoneal injection (i.p.) for 4 weeks according to a previously established protocol (29). On the final day of treatment, the last SCO injection was administered 30 min before the Y-maze test. Immediately after completion of the Y-maze test, mice were euthanized, and brain tissues were collected for subsequent biochemical and molecular analyses. Both hesperetin and SCO were purchased from Sigma-Aldrich (St. Louis, MO, USA). Body weight and food intake were recorded every 3 days. All experimental procedures were approved by the Animal Care and Use Committee of Chonnam National University (CNU IACUC-YB-2023-79).

### Y-maze test

To assess the short-term memory-enhancing effects of hesperetin in an SCO-induced mouse model of cognitive dysfunction, a Y-maze test was conducted to measure spontaneous alternation behavior. The Y-maze apparatus, constructed from white plastic, consisted of three arms (A, B, and C). Each arm measured 35 cm in length, 10 cm in height, and 5 cm in width. During testing, each mouse was randomly placed at the end of one arm, and its movements were recorded over an 8-min session. The primary behavioral parameter was the sequence of arm entries. A correct alternation was defined as successive entries into all three arms (e.g. ABC or ACB), while repeated entries (e.g. ABB or ACC) were excluded from the alternation count. Spontaneous alternation percentage was calculated using the following formula: [number of spontaneous alternations / (total arm entries – 2)] × 100.

### Histopathological examination of brain tissues

Hematoxylin and eosin (H&E) staining was performed to evaluate morphological changes in brain tissue. Brain samples from representative mice in each group were fixed in 10% neutral-buffered formalin pH 7.0, rinsed with phosphate-buffered saline, and embedded in paraffin. Tissue sections approximately 4–5 µm thick were cut, mounted on slides, and stained with H&E. Histological analysis was conducted under a light microscope at 400 × magnification.

### Biochemical analysis

To assess the effects of hesperetin on lipid metabolism, levels of alanine aminotransferase (ALT), aspartate aminotransferase (AST), total cholesterol (TC), triglyceride (TG), high-density lipoprotein cholesterol (HDL-C), and low-density lipoprotein cholesterol (LDL-C) were measured. Levels of ALT, AST, TG, TC, and HDL-C were measured using commercial assay kits (Asan Pharmaceutical, Seoul, South Korea). LDL-C was calculated using the following formula: LDL-C = TC - (HDL-C + TG/5).

### Measurement of AChE activity

To investigate the cognitive-enhancing effects of hesperetin, the activity of AChE, a key enzyme involved in cholinergic neurotransmission, was measured in serum. AChE activity was assessed using a colorimetric AChE assay kit (Abcam, Cambridge, UK), according to the manufacturer’s instructions. Absorbance was measured at 410 nm using a microplate reader, and enzyme activity was determined based on the standard curve provided with the kit.

### Measurement of malondialdehyde levels

Malondialdehyde (MDA) levels were measured to assess lipid peroxidation in brain tissue. MDA, a biomarker of oxidative lipid damage, was quantified using enzyme-linked immunosorbent assay (ELISA) kits (Elabscience, Houston, TX, USA). Brain tissues were homogenized in ice-cold phosphate buffer and centrifuged at 3,000 × g for 15 min. All procedures were conducted strictly according to the manufacturer’s instructions.

### Immunoblotting analysis

Immunoblotting was performed to evaluate the expression of proteins involved in inflammation, apoptosis, and the TrkB/AKT/CREB/BDNF signaling pathway. Whole-cell lysates were extracted using RIPA buffer (Biosesang, Sungnam, Korea) enhanced with Halt™ protease and phosphatase inhibitor cocktail (Thermo Fisher Scientific, Waltham, MA, USA). Nuclear lysates were prepared using a nuclear extraction buffer containing 20 mM HEPES, 0.4 mM NaCl, 1 mM EDTA, 1 mM EGTA, 1 mM dithiothreitol, and 1 mM PMSF, supplemented with 10% NP-40. Protein concentrations were determined using a BCA protein assay kit (Pierce, IL, USA, according to the manufacturer’s guidelines). Proteins (20 μg) were separated by SDS-PAGE and transferred to a nitrocellulose membrane (Invitrogen, Waltham, MA, USA). Membranes were blocked for 2 h in a solution of 10 mM Tris-HCl (pH 7.5), 150 mM NaCl, 0.1% Tween 20, and 5% non-fat dry milk and incubated with primary antibodies for 2 h. After washing, membranes were incubated with a conjugated secondary antibody for an additional 2 h. Protein bands were visualized using western blotting luminol reagent (Santa Cruz Biotechnology, Dallas, TX, USA) and imaged with the ChemiDoc XRS+ Imaging System (BioRad, Hercules, CA, USA). Densitometric analysis was performed using ImageJ software, and protein expression levels were normalized to β-actin.

### Quantitative polymerase chain reaction analysis

Quantitative polymerase chain reaction (qPCR) was used to analyze mRNA expression of genes related to inflammation, apoptosis, and the TrkB/AKT/CREB/BDNF signaling pathway. Total RNA was extracted using TRIzol reagent, following the manufacturer’s instructions (Thermo Fisher Scientific, Waltham, MA, USA). The RNA concentration and purity were verified by absorbance measurements at 260 and 280 nm using a NanoDrop 2000 spectrophotometer (Thermo Fisher Scientific, Waltham, MA, USA). First-strand cDNA was synthesized from 1 μg of total RNA using an Omniscript RT kit (QIAGEN, Hilden, Germany). qPCR was conducted using SYBR Green dye on a CFX96 Touch Real-Time PCR Detection System (Bio-Rad, CA, USA), with all reactions performed in triplicate. Relative gene expression was calculated using the 2^–∆∆CT^ method, with normalization to β-actin.

### ELISA

Serum samples were collected immediately after euthanasia and stored at –80°C until analysis. Levels of the proinflammatory cytokines tumor necrosis factor-α (TNF-α) and interleukin 6 (IL-6) were quantified using ELISA kits (Elabscience, Houston, TX, USA), in accordance with the manufacturer’s instructions. Concentrations were calculated from a standard curve.

### Histone extraction and purification

To investigate histone acetylation and methylation patterns related to epigenetic regulation, core histones were extracted from brain tissue using a Histone Purification Mini Kit (Active Motif, Carlsbad, CA, USA) per the manufacturer’s instructions. Tissues were homogenized in ice-cold extraction buffer using a Dounce homogenizer and rotating platform. The homogenate was centrifuged at 16,000 × g for 5 min at 4°C, and the supernatant was neutralized to pH 8.0. After equilibrating spin columns with equilibration buffer, crude histone extracts were loaded onto the columns. The columns were washed three times, and histones were eluted with elution buffer. Eluted proteins were precipitated overnight at 4°C using 4% perchloric acid (PCA) and centrifuged at 16,000 × g for 1 h at 4°C. The resulting pellet was washed with acetone and PCA, and purified histones were resuspended in diethyl pyrocarbonate-treated water, quantified, and prepared for further analysis.

### Statistical analysis

All experiments were conducted in triplicate, and data are presented as mean ± standard deviation (SD). Statistical significance among groups was evaluated using one-way ANOVA, followed by Duncan’s multiple range test, using SPSS version 25.0 (IBM SPSS Institute, Chicago, IL, USA). Significance thresholds are detailed in the figure legends, with *P* < 0.05 considered statistically significant.

## Results

### Effect of hesperetin on SCO-induced memory loss in the Y-maze test

The Y-maze test, a well-established method for evaluating spatial memory and cognitive function, was used to assess the effects of hesperetin on SCO-induced memory impairment. In this test, mice are allowed to explore all three arms of the maze, leveraging their innate curiosity to investigate novel environments. Spontaneous alternation behavior, which reflects memory function, is widely accepted as an indicator of spatial working memory (30). As shown in Fig. 1, the percentage of spontaneous alternation was significantly reduced in the SCO group (38.98%) compared with the CON group (79.34%) (*P* < 0.05), indicating impaired memory performance. However, oral administration of hesperetin at doses of 25 mg/kg and 50 mg/kg in the SCOH25 and SCOH50 groups significantly improved spontaneous alternation to 67.09 and 80.17%, respectively (*P* < 0.05). These findings, indicate that hesperetin effectively mitigates SCO-induced deficits in spatial memory.

**Fig. 1:**
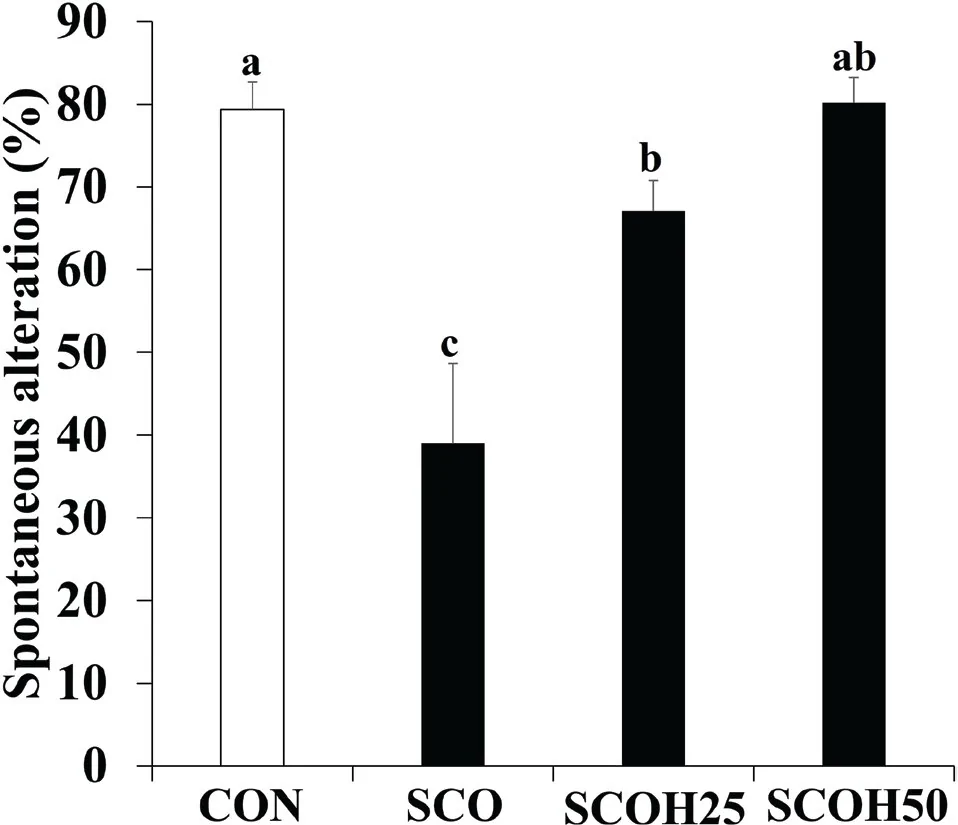
Effect of hesperetin on scopolamine-induced memory impairments in mice assessed by the Y-maze test. Mice were pretreated with hesperetin (25 or 50 mg/kg) 30 min before scopolamine administration (1 mg/kg). Spontaneous alternation behavior was assessed during an 8-min Y-maze session. Data are presented as mean ± standard deviation (SD; *n* = 10). Different letters indicate statistically significant differences (*P* < 0.05), as determined by Duncan’s multiple range test. CON: control; SCO: scopolamine 1 mg/kg; SCOH25: scopolamine + hesperetin (25 mg/kg/day); SCOH50: scopolamine + hesperetin (50 mg/kg/day); SD: standard deviation.

### Hesperetin administration ameliorated SCO-induced brain injury in C57BL/6 mice

The neuroprotective effects of hesperetin were further confirmed through histological examination of brain tissue using H&E staining. In the CON group, normal neuronal morphology and intact cellular architecture were observed in both the hippocampus and cerebral cortex (Fig. 2). In contrast, the SCO group exhibited pronounced neuronal damage in the hippocampus characterized by nuclear condensation and disrupted cytoarchitecture. Hesperetin treatment markedly attenuated these pathological changes. These histological findings support the role of hesperetin in protecting against SCO-induced neurodegeneration.

**Fig. 2:**
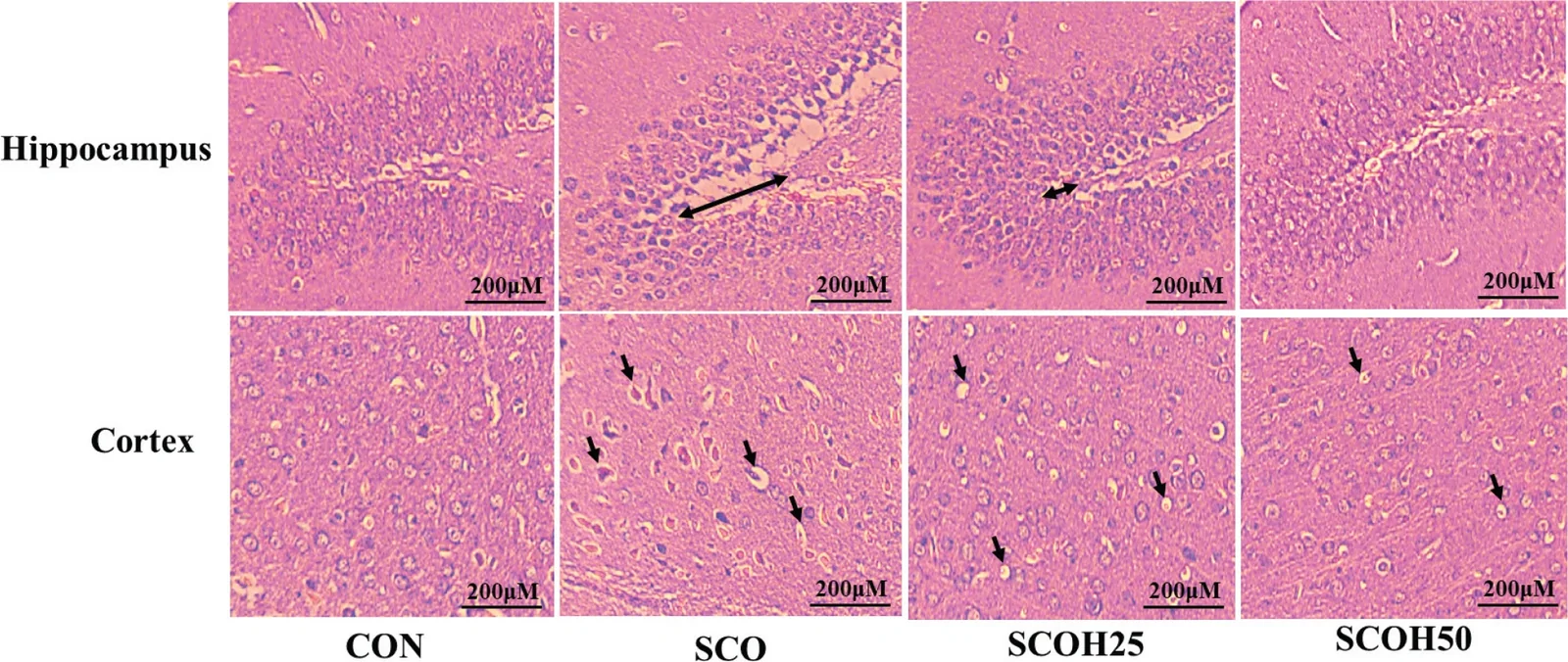
Effect of hesperetin administration on mouse brain histology assessed by hematoxylin and eosin (H&E) staining. Representative photomicrographs of H&E-stained brain sections (magnification, 400×) from formalin-fixed, paraffin-embedded tissue. CON: control; SCO: scopolamine 1 mg/kg; SCOH25: scopolamine + hesperetin (25 mg/kg/day); SCOH50: scopolamine + hesperetin (50 mg/kg/day).

### Hesperetin improved serum lipid profiles in SCO-treated mice

To evaluate the impact of hesperetin on liver function and lipid metabolism, serum levels of ALT and AST, TC, and TG were measured. As shown in Table 1, the SCO group exhibited elevated AST and ALT levels compared with the CON group although these increases were not statistically significant. Hesperetin-treated groups (SCOH25 and SCOH50) showed reductions in both AST and ALT levels relative to the SCO group. Lipid profile analysis revealed significantly higher TC, TG, and LDL-C levels in the SCO group compared with the CON (*P* < 0.05), indicating dyslipidemia associated with SCO treatment. Hesperetin administration at 50 mg/kg significantly decreased TG and TC levels compared with the SCO group (*P* < 0.05). Furthermore, LDL-C levels were significantly reduced in both SCOH25 and SCOH50 groups, while HDL-C levels were significantly higher in the SCO group than in the CON (*P* < 0.05). Collectively, these results suggest that hesperetin may exert hepatoprotective effects and improve lipid metabolism in the context of SCO-induced cognitive dysfunction.

**Table 1:** Effect of hesperetin administration on serum metabolic parameters

Parameters	CON	SCO	SCOH25	SCOH50
AST	26.21 ± 2.49	33.37 ± 6.40	29.90 ± 4.21	29.64 ± 2.96
ALT	17.78 ± 2.91	21.73 ± 1.79	20.28 ± 1.72	19.49 ± 1.59
TC (mg/dL)	60.94 ± 5.38^c^	92.94 ± 5.87^a^	80.46 ± 2.22^b^	66.10 ± 4.08^bc^
TG (mg/dL)	55.94 ± 4.12^c^	89.72 ± 4.12^a^	75.05 ± 4.71^b^	61.29 ± 3.25^c^
HDL-C (mg/dL)	105.82 ± 7.32^a^	56.48 ± 8.08^c^	69.12 ± 12.59^b^	74.36 ± 7.59^b^
LDL-C (mg/dL)	28.59 ± 5.68^c^	63.70 ± 7.51^a^	51.63 ± 3.84^b^	36.96 ± 3.44^bc^

Data are presented as mean ± standard deviation (SD; *n* = 10). Values with different superscript letters within a row indicate statistically significant differences (*P* < 0.05), as determined by Duncan’s multiple range test.

CON: control; SCO: scopolamine 1 mg/kg; SCOH25: scopolamine + hesperetin (25 mg/kg/day); SCOH50: scopolamine + hesperetin (50 mg/kg/day); AST: aspartate aminotransferase; ALT: alanine aminotransferase; TC: total cholesterol; TG: triglycerides; HDL-C: high-density lipoprotein cholesterol; LDL-C: low-density lipoprotein cholesterol; SD: standard deviation.

### Effect of hesperetin on brain AChE activity in SCO-treated mice

To explore the effects of hesperetin on cholinergic function, brain AChE was measured using a colorimetric AChE assay. As shown in Fig. 3A, AChE activity was significantly elevated in the SCO group (563.1 μU/mL) compared with the CON group (256.9 μU/mL) (*P* < 0.05). Treatment with hesperetin significantly reduced AChE activity to 445.8 μU/mL (SCOH25) and 326.2 μU/mL (SCOH50) (*P* < 0.05), indicating a dose-dependent inhibition of AChE. These results suggest that hesperetin may restore cholinergic balance by reducing AChE activity, thereby contributing to the alleviation of SCO-induced cognitive impairment.

**Fig. 3:**
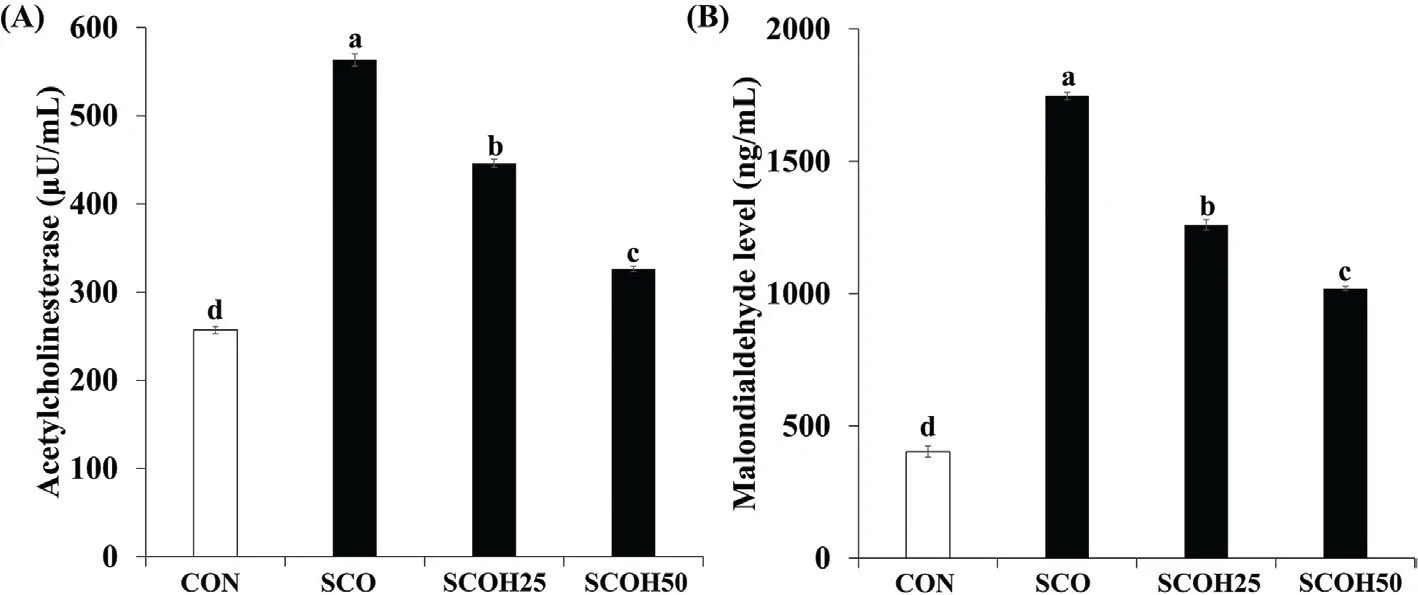
Effect of hesperetin on AChE activity and MDA levels in the brains of scopolamine-induced memory-impaired mice. (A) AChE activity was measured using an AChE assay. (B) MDA levels were measured using an MDA assay. All experiments were performed in at least triplicate, and results are presented as mean ± SD. Different letters indicate statistically significant differences (*P* < 0.05), as determined by Duncan’s multiple range test. CON: control; SCO: scopolamine 1 mg/kg; SCOH25: Scopolamine + hesperetin (25 mg/kg/day); SCOH50: scopolamine + hesperetin (50 mg/kg/day); AChE: acetylcholinesterase; MDA: malondialdehyde; SD: standard deviation.

### Hesperetin suppresses SCO-induced MDA level

MDA, a byproduct of lipid peroxidation, is markedly elevated in mice with SCO-induced cognitive impairment (31). To evaluate the effect of hesperetin on oxidative stress, MDA levels in brain tissue were quantified using the MDA assay. As shown in Fig. 3B, MDA concentrations were significantly higher in the SCO group (1746.7 ng/mL) compared with the CON group (402.7 ng/mL) (*P* < 0.05). Notably, hesperetin administration reduced MDA levels in a dose-dependent manner, with the SCOH25 and SCOH50 groups showing values of 1259.1 and 1019.3 ng/mL, respectively (*P* < 0.05).

### Effects of hesperetin on pro-inflammatory cytokine release and related gene expression via the NF-κB pathway in SCO-treated mice brain

SCO induces neuroinflammation and cognitive dysfunction by activating NF-κB signaling and proinflammatory cytokines (11). To investigate the anti-inflammatory potential of hesperetin, we assessed cytokine release and NF-κB pathway activation. ELISA results revealed significantly elevated levels of TNF-α and IL-6 in the SCO group, which were attenuated following hesperetin treatment (Fig. 4A and B). Furthermore, western blot analysis demonstrated a dose-dependent reduction in the expression of inflammatory proteins cyclooxygenase-2 (COX-2), IL-6, iNOS, and TNF-α in the hesperetin-treated group (Fig. 4C–G). Hesperetin also significantly decreased the levels of phosphorylated IκBα and NF-κB (Fig. 4K, M, and N) and suppressed NF-κB mRNA expression compared with the SCO group (*P* < 0.05) (Fig. 4L).

**Fig. 4:**
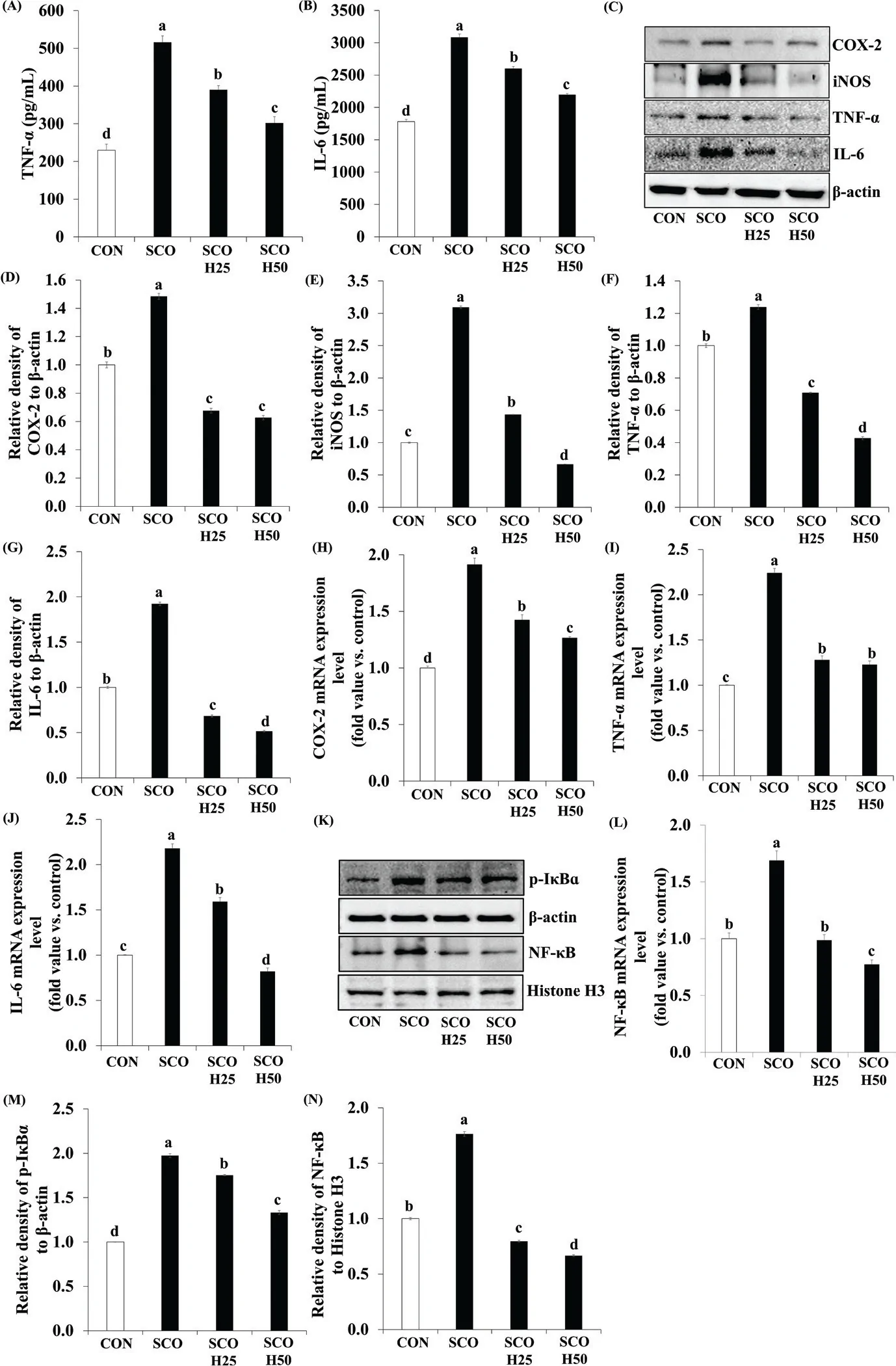
Effect of hesperetin on inflammatory cytokine expression and NF-κB activation in the brains of scopolamine-induced memory-impaired mice. (A and B) IL-6 and TNF-α secretion levels were measured using an ELISA kit: (A) TNF-α; (B) IL-6. (C) Protein expression levels of TNF-α, IL-6, iNOS, and COX-2 were assessed using Western blotting. (D–G) Protein band densities were normalized to β-actin using ImageJ software. (H–J) mRNA levels of COX-2, TNF-α, and IL-6 were quantified and normalized to β-actin mRNA. Expression values are presented relative to the CON group, which was arbitrarily defined as 1. (K) Protein levels of phosphorylated NF-kappa-B inhibitor alpha (p-IκBα) and nuclear factor-kappa B (NF-κB) were analyzed by western blotting. Equal protein loading was confirmed by stripping and reprobing for histone H3. (L) NF-κB mRNA expression was quantified using the 2^–ΔΔCT^ method, normalized to β-actin. (M and N) Protein band densities were quantified using ImageJ software and normalized to β-actin. All experiments were conducted in at least triplicate. Data are presented as mean ± SD and analyzed using the 2^–ΔΔCT^ method. Different letters indicate statistically significant differences (*P* < 0.05), as determined by Duncan’s multiple range test. CON: control; SCO: scopolamine 1 mg/kg; SCOH25: scopolamine + hesperetin (25 mg/kg/day); SCOH50: scopolamine + hesperetin (50 mg/kg/day); TNF-α: tumor necrosis factor-α; IL-6: interleukin 6; COX-2: cyclooxygenase-2; iNOS: inducible nitric oxide synthase; ELISA: enzyme-linked immunosorbent assay; p-IκBα: phosphorylated NF-kappa-B inhibitor alpha; NF-κB: nuclear factor-kappa B; SD: standard deviation.

### Effects of hesperetin on apoptosis signaling in SCO-treated mice brain

SCO-induced cognitive dysfunction is associated with neuronal apoptosis (12). To assess the anti-apoptotic properties of hesperetin, we examined the expression of apoptosis-related proteins and genes using western blotting and qPCR. Hesperetin enhanced caspase-3 protein expression in brain tissue (Fig. 5A), indicating activation of downstream apoptotic signaling. While SCO increased the expression of the pro-apoptotic Bcl-2–associated X protein (Bax), hesperetin treatment decreased Bax levels and elevated B-cell leukemia/lymphoma 2 protein (Bcl-2), an anti-apoptotic marker, particularly in the SCOH50 group (Fig. 5A, C, and D). These changes led to a significant reduction in the Bax/Bcl-2 ratio in the SCOH50 group compared with the SCO group (*P* < 0.05) (Fig. 5B), suggesting that hesperetin confers neuroprotection by modulating apoptosis.

**Fig. 5:**
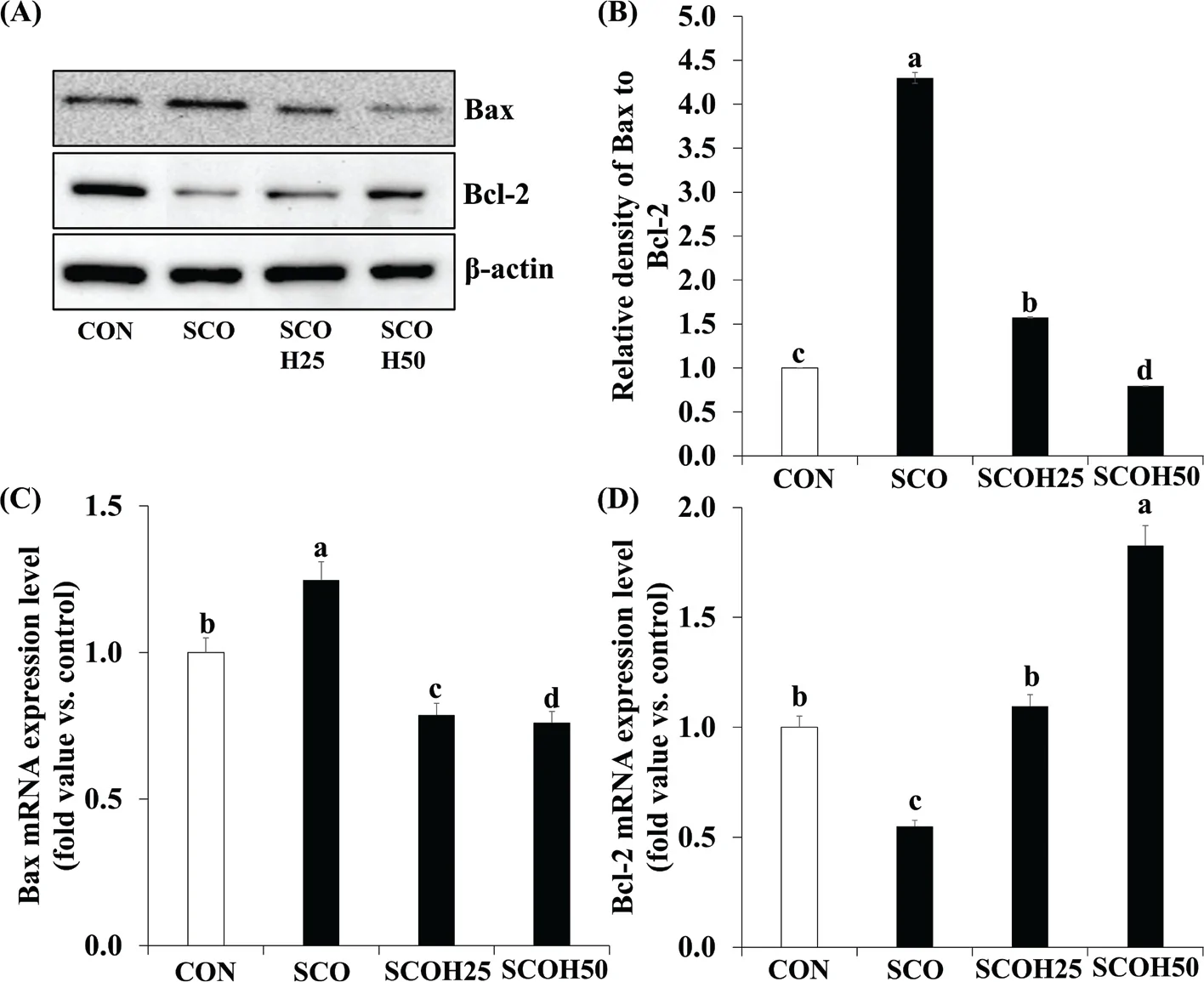
Effects of hesperetin on apoptosis protein expression in the brains of scopolamine-induced memory-impaired mice. (A) Protein expression levels of Bax and Bcl-2 were assessed by immunoblotting. (B) Bax/Bcl-2 ratios were calculated, with band densities normalized to β-actin, using ImageJ software. (C and D) Relative mRNA expression levels of Bax and Bcl-2 were analyzed following normalization to β-actin mRNA. Expression values are presented relative to the control group, arbitrarily set to 1. Experiments were conducted in triplicate, and results are presented as mean ± SD. Data were analyzed using the 2^–ΔΔCT^ method. Different letters indicate statistically significant differences (*P* < 0.05), as determined by Duncan’s multiple range test. CON: control; SCO: scopolamine 1 mg/kg; SCOH25: scopolamine + hesperetin (25 mg/kg/day); SCOH50: scopolamine + hesperetin (50 mg/kg/day); Bax: Bcl2-associated X protein; Bcl-2: B-cell lymphoma 2; SD: standard deviation.

### Hesperetin improves memory loss in SCO-treated mice by regulating the TrkB/BDNF/CREB signaling pathway

To elucidate the molecular mechanisms underlying the cognitive benefits of hesperetin, we investigated its effects on the TrkB/BDNF/CREB signaling axis in the brains of SCO-treated mice. Western blotting and qPCR analyses revealed that SCO significantly reduced the expression of TrkB, BDNF, CREB, and phosphorylated protein kinase B (AKT) (Fig. 6). In contrast, hesperetin treatment restored the expression of TrkB and BDNF and significantly increased the phosphorylation of CREB and AKT. These findings suggest that hesperetin enhances memory and learning by activating the TrkB/AKT/BDNF/CREB signaling pathway.

**Fig. 6:**
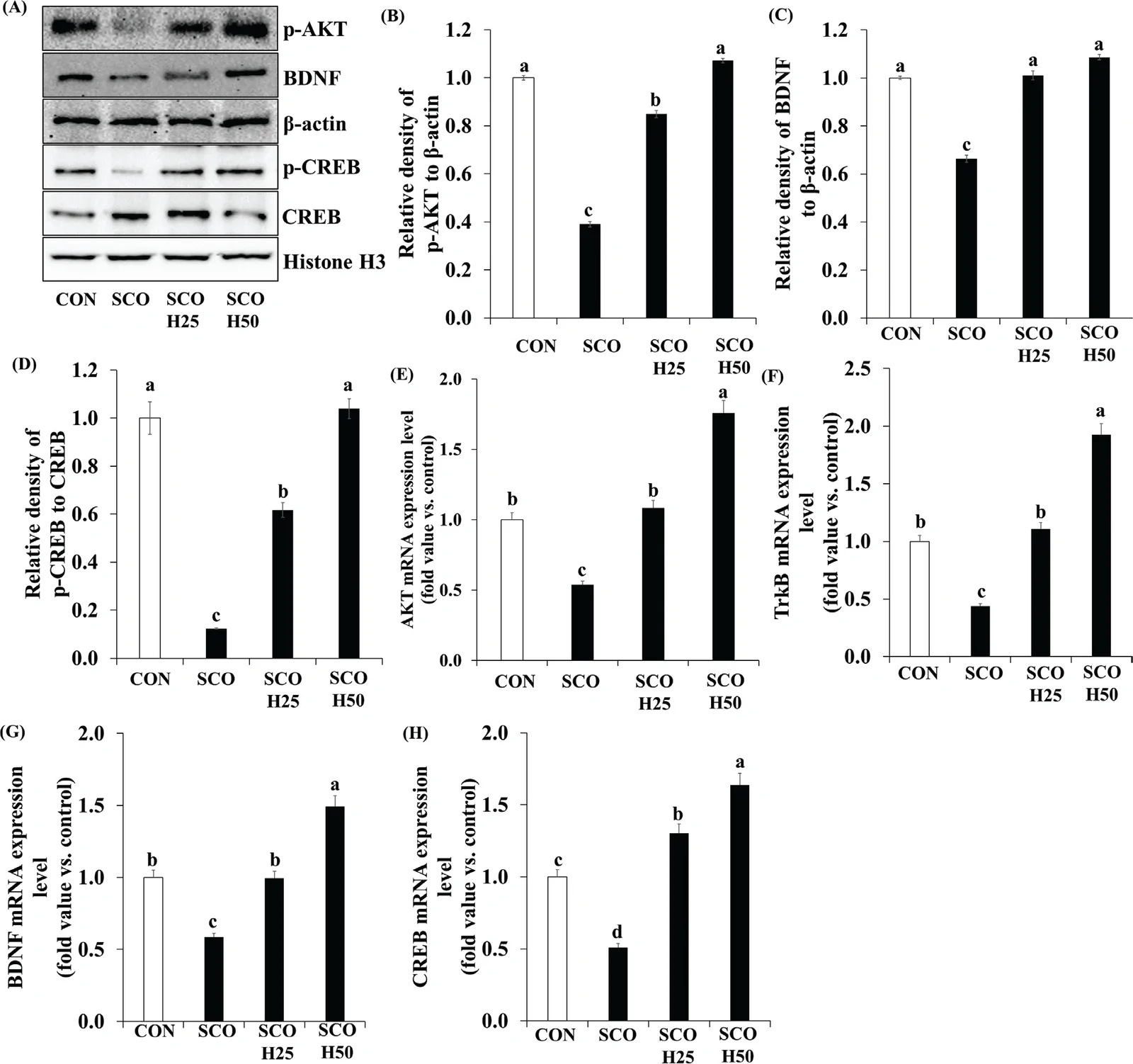
Hesperetin regulates the TrkB/CREB/BDNF pathway in the brains of scopolamine-induced memory-impaired mice. (A) Protein expression levels of p-AKT, BDNF, p-CREB, and CREB were assessed by immunoblotting. (B–D) Band densities were quantified and normalized to β-actin using ImageJ software. (E–H) mRNA expression levels of AKT, TrkB, BDNF, and CREB were measured in brain tissue, normalized to β-actin mRNA. Expression values are presented relative to the CON group, arbitrarily set to 1. Experiments were performed in triplicate, and results are presented as mean ± SD. Statistical analysis was conducted using the 2^–ΔΔCT^ method. Different letters indicate statistically significant differences (*P* < 0.05), as determined by Duncan’s multiple range test. CON: control; SCO: scopolamine 1 mg/kg; SCOH25: scopolamine + hesperetin (25 mg/kg/day); SCOH50: scopolamine + hesperetin (50 mg/kg/day); AKT: protein kinase B; CREB: cAMP response element-binding protein; BDNF: brain-derived neurotrophic factor; SD: standard deviation.

### Hesperetin administration reversed SCO-induced changes in histone methylation and acetylation and related gene expression

Histone acetylation and methylation are important epigenetic mechanisms involved in memory formation, synaptic plasticity, and cognitive function regulation. SCO reduces the level of histone acetylation, thereby downregulating the expression of memory-related genes such as *BDNF* and *CREB*, thereby causing cognitive impairment (8). As depicted in Fig. 7A and B, H3K9 and H3K27 acetylation levels decreased in the brains of SCO-treated mice. In contrast, these acetylation levels were elevated in the SCOH50 group compared with the SCO group. Similarly, H3K4 and H3K27 dimethylation levels were reduced in the SCO group but increased in the SCOH50 group. We measured the expression of HDAC2, HDAC4, and SIRT1, key regulators of histone methylation and acetylation, in mouse brain tissue. Compared with the CON, the SCO group exhibited increased expression of HDAC2 and HDAC4, along with decreased SIRT1 expression (Fig. 7C–F). Conversely, the SCOH50 group showed reduced HDAC2 and HDAC4 expression and elevated SIRT1 expression compared with the SCO group.

**Fig. 7:**
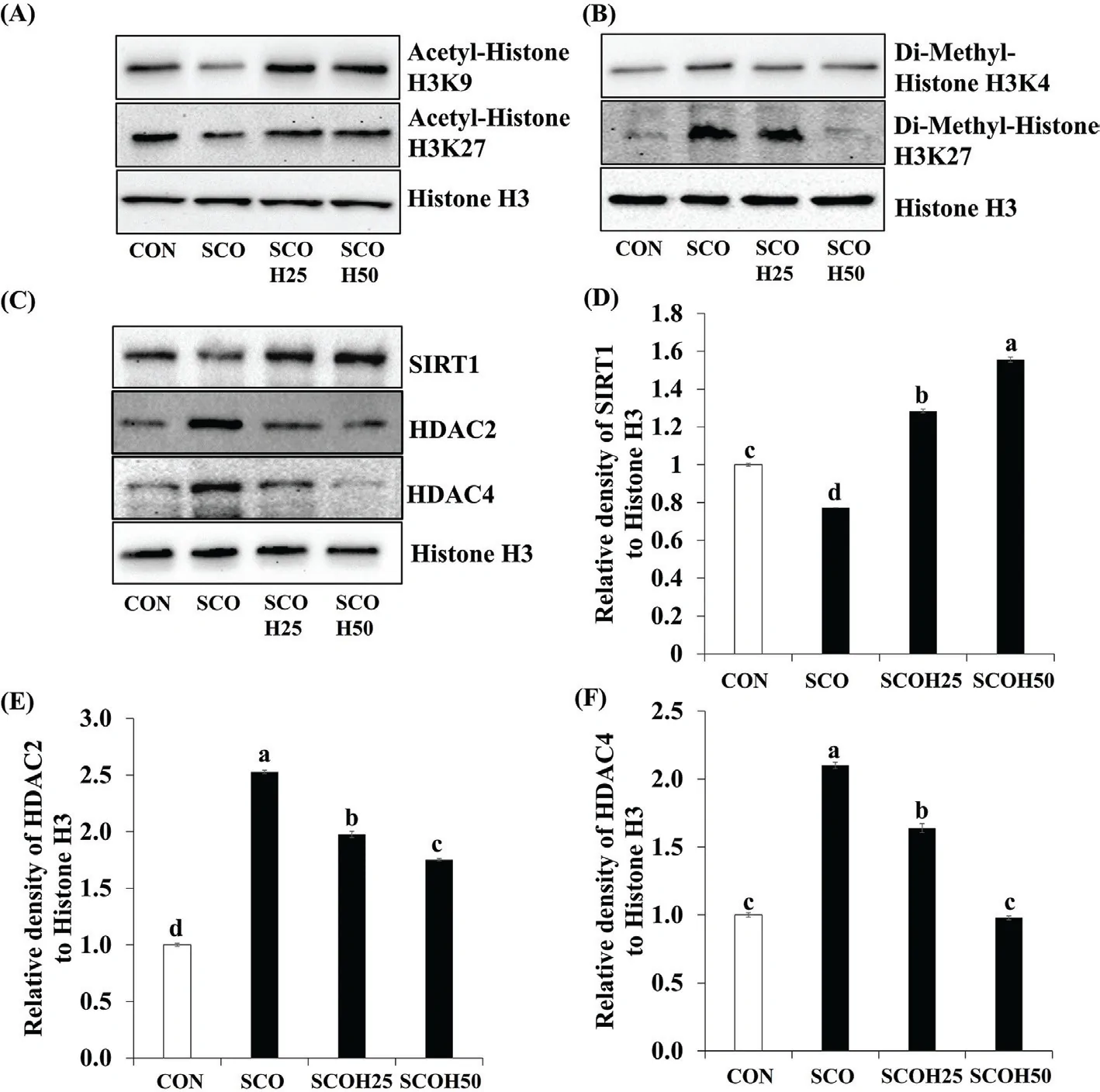
Hesperetin regulates histone modification and associated protein expression in the brains of scopolamine-induced memory-impaired mice. (A) Protein levels of acetylated histones H3K9 and H3K27 were assessed by immunoblotting. (B) Levels of H3K9 and H3K27 dimethylation were measured by immunoblotting, with equal loading of protein ensured by stripping the immunoblot and reprobing it for histone H3. (C)Western blotting was used to assess the expression levels of SIRT1, HDAC2, and HDAC4. (D–F) Protein band densities were quantified and normalized to β-actin using ImageJ software. Equal protein loading was confirmed by stripping the blot and reprobing for histone H3. CON: control; SCO: scopolamine 1 mg/kg; SCOH25: scopolamine + hesperetin (25 mg/kg/day); SCOH50: scopolamine + hesperetin (50 mg/kg/day); SIRT1: sirtuin 1; HDAC: histone deacetylases; SD: standard deviation.

## Discussion

Neurodegenerative diseases are clinical syndromes characterized by progressive memory loss, cognitive impairment, and extensive structural abnormalities in the brain. These conditions result from structural and functional impairments of neurons (2). Cognitive impairment, one of the primary symptoms, becomes increasingly prevalent with age (1). Its pathogenesis involves multiple pathological factors, including increased reactive oxygen species (ROS) generation, ACh depletion, elevated oxidative stress, neuroinflammation, and neuronal loss (3). Current treatments for cognitive impairment, such as cholinesterase inhibitors and N-methyl-D-aspartate (NMDA) receptor antagonists, offer limited efficacy and are frequently associated with adverse effects, including vomiting and hypertension (4). Due to these limitations, there is growing global interest in safer, plant-derived alternatives (23). Medicinal herbs and natural products play a vital role in safeguarding against cognitive decline. Hesperetin, a phenolic compound abundantly present in oranges, grapefruits, and grape skins, possesses anti-hyperlipidemic, anti-diabetic, and anti-inflammatory properties (23). This study investigated the neuroprotective effects of hesperetin against SCO-induced cognitive deficits in C57BL/6 mice.

SCO, an anticholinergic drug, is also known to induce cognitive side effects such as memory impairment; therefore, it is widely used to model cognitive dysfunction in mice (5, 9). The C57BL/6 mouse strain is particularly susceptible to SCO-induced cholinergic dysfunction, neuroinflammation, and memory deficits, making it a preferred model for such studies (32). The Y-maze test, an SCO-induced cognitive impairment behavioral experiment, is a widely used method to evaluate hippocampus-dependent working memory based on voluntary alternating behavior without prior training or aversive stimuli (28). Therefore, the Y-maze was selected in the present study to sensitively evaluate working memory impairment induced by repeated SCO administration while minimizing experimental stress. Our findings demonstrated that hesperetin significantly improved alternation behavior in a concentration-dependent manner compared with the SCO. Similar cognitive enhancements in SCO-treated mice have been reported with other phytochemicals, such as glycyrrhizic acid (33).

The brain is a lipid-rich organ, and lipid homeostasis is essential for maintaining neuronal membrane integrity, synaptic plasticity, and normal neuronal function. Increasing evidence indicates that dyslipidemia contributes to cognitive decline by promoting oxidative stress, neuroinflammation, cerebrovascular dysfunction, and impaired neuronal signaling (15, 34).

SCO blocks ACh receptors, impairs autonomic nervous system function and alters lipid metabolism (5). Hyperlipidemia, characterized by elevated cholesterol levels, has been identified as a potential risk factor for the progression of neurodegenerative diseases (5). Recent research indicates that increased ROS generated from SCO promotes intracellular cholesterol accumulation through upregulation of NF-κB signaling (11). In addition, HDL levels have been associated with regulated cognitive function in neurodegenerative diseases, whereas elevated LDL levels correlate with increased disease risk. Drugs targeting cholesterol synthesis have also been shown to reduce amyloid β deposition (35). A recent *in vivo* study demonstrated that SCO may induce hyperlipidemia by elevating serum levels of TG, LDL cholesterol, and TC (35). Consistent with these studies, our results demonstrated that SCO significantly increased TC, TG, and LDL levels while decreasing HDL levels. Conversely, hesperetin administration reversed these changes, lowering TC, TG, and LDL levels and elevating HDL levels in the treated mice. These findings suggest that improvement in lipid metabolism may contribute to the observed improvement in cognitive function. Our results further indicate that improved lipid metabolism is associated with cognitive improvement, suggesting that the beneficial effects of hesperetin are at least partially mediated through the regulation of lipid metabolism.

Oxidative stress induces alterations in neuronal cells, as demonstrated by increased lipid peroxidation and diminished activity of antioxidant enzymes, particularly in neurodegenerative diseases (36). MDA, a terminal product of lipid peroxidation, forms when polyunsaturated fatty acids undergo oxidative degradation due to excessive ROS. This lipid damage compromises membrane permeability, fluidity, and receptor function, thereby impairing synaptic transmission and leading to neuronal injury. These changes contribute to memory loss and cognitive decline through key neurodegenerative mechanisms (31). Animal studies have demonstrated that SCO elevates MDA levels in the brain, indicating oxidative stress (31). Natural compounds such as *Terminalia chebula* extract and imperatorine have shown efficacy in reducing MDA levels and mitigating cognitive impairment in SCO-treated models (37). Consistent with these findings, our data indicate that SCO significantly elevates MDA levels, while hesperetin administration mitigates this effect by reducing MDA concentration in mice with SCO-induced cognitive impairment.

Neurodegenerative diseases are characterized by reductions in ACh levels and neuronal loss, particularly in the cholinergic system of the hippocampus, leading to memory impairment (38). In dementia, increased AChE activity and reduced ACh levels correlate with memory decline (39). These cholinergic deficits reflect underlying neuropathological changes and are consistently associated with the severity of cognitive impairment (40). In animal studies, SCO has been shown to decrease ACh levels and increase AChE activity, contributing to cognitive impairment and neuronal cell death (39). Moreover, elevated AChE activity has been linked to increased ROS production in the brain (36). Our results confirm that hesperetin administration inhibits AChE activity in mice with SCO-induced cognitive impairment. Similar inhibitory effects have been reported with natural compounds such as bergapten and *Angelica keiskei* extracts in SCO-treated mice (38, 41).

In dementia, activated microglia and astrocytes produce ROS, chemokines, cytokines, and proinflammatory enzymes, contributing to memory impairments (36). ROS-induced oxidative stress is closely associated with neuroinflammation, which accelerates the progression of neurodegenerative diseases (42). SCO administration increases levels of IL-1β, IL-12, and TNF-α in the hippocampus of mice (43). NF-κB, a key regulator of inflammatory cytokines, is highly activated in neuroinflammatory conditions. Upon activation, NF-κB translocates from the cytoplasm to the nucleus, promoting the expression of proinflammatory genes (11). Although inflammation can promote tissue repair through immune cells and inflammatory mediators, excessive inflammatory activity aggravates many disease processes (43). Our results confirmed that SCO upregulated the expression of NF-κB, TNF-α, IL-6 and COX-2 in mice with cognitive impairment, indicating an activated inflammatory response. Conversely, hesperetin treatment downregulated these inflammatory markers. Supporting these findings, recent studies have demonstrated that dietary compounds such as quercetin, vanillin, and friedelin inhibit inflammatory cytokines associated with cognitive impairment in both *in vivo* and clinical settings (42, 44), suggesting that hesperetin may similarly suppress SCO-induced neuroinflammation by modulating NF-κB signaling.

ROS, as key mediators of oxidative stress, initiate apoptosis and cause severe damage to proteins and DNA, impairing neuronal structure and function (36). While apoptosis under normal conditions is essential for development, homeostasis, and protection against cellular damage and mutation, excessive apoptosis disrupts homeostasis and contributes to disease progression (13). In patients with AD, increased oxidative stress in the brain leads to irreversible cell death and structural brain damage (45). The Bcl-2 protein family, which includes pro-apoptotic members such as Bad and Bax and anti-apoptotic members such as Bcl-2 and Bcl-xL, plays a pivotal role in mitochondrial-mediated apoptosis (46). Upon oxidative stress, Bax translocates to the mitochondrial membrane, facilitating the release of cytochrome C into the cytoplasm, which subsequently activates caspase-3 neuronal cell death. In contrast, Bcl-2 inhibits apoptosis by preventing this release (47). The Bax/Bcl-2 ratio is a crucial indicator of the propensity of a cell for survival or death (46). SCO-induced apoptosis upregulates Bax expression and downregulates Bcl-2 expression, thereby promoting neuronal loss (46). Consistent with previous findings, extracts from *Cyperus esculentus L*. and apigenin have been shown to downregulate Bax and upregulate Bcl-2 expression in SCO-induced cognitive impairment in mice (47). Similarly, our results demonstrated that hesperetin treatment reduced the Bax/Bcl-2 ratio in mice with SCO-induced cognitive dysfunction.

The TrkB/AKT/BDNF/CREB signaling pathway has been identified as a key regulator of hippocampal neurogenesis in both rodent models of memory impairment and patients with dementia (8). Activation of TrkB receptors through the PI3K/AKT pathway is essential for neural development and neuronal survival (48). These receptors are involved in various physiological and pathological processes in the nerves and other tissues. Impaired TrkB signaling is a significant factor in the pathogenesis of neurodegenerative diseases (49).

In the context of neuroprotection, activation of the TrkB receptor enhances the PI3K/AKT signaling pathway, which subsequently promotes CREB phosphorylation and regulates BDNF expression, thereby contributing to improved cognitive function and learning capacity. As a key transcription factor, CREB plays a central role in neuronal survival, synaptic plasticity, and memory formation (8). Its activation is closely associated with neuroprotective effects, partly through the regulation of downstream targets such as Bcl-2, which mitigates ROS-induced cytotoxicity (46). Conversely, reduced CREB phosphorylation has been implicated in the pathogenesis of several neurodegenerative disorders, including AD, Huntington’s disease, and Parkinson’s disease (8).

*BDNF*, a CREB target gene, is crucial for neuronal survival, differentiation, and the formation and regulation of synaptic networks underlying memory and learning (18). Beyond its effects on cholinergic transmission, SCO has been shown to suppress the expression of AKT/BDNF/CREB, key mediators of long-term memory formation in the central nervous system. This downregulation is particularly evident in neurodegenerative disease models (49). Recent evidence supports the therapeutic potential of natural compounds such as ginsenoside Rg1, artemisinin, and oridonin in modulating this pathway to counteract neurodegeneration (49). For instance, *Enteromorpha prolifera* extract enhances memory performance by activating the AKT/TrkB pathway in SCO-treated mice (48). Consistent with these findings, our results demonstrated that hesperetin treatment significantly upregulated *TrkB*, *BDNF*, *CREB*, and *AKT* expression in SCO-induced cognitive impairment in mice, highlighting its potential to restore TrkB/AKT/CREB/BDNF signaling and ameliorate cognitive decline associated with neurodegenerative conditions.

Epigenetic mechanisms play a pivotal role in the cellular damage underlying brain aging (19). Research indicates that reduced histone acetylation and elevated histone methylation contribute substantially to cognitive impairment. Specifically, increased levels of H3K4me2 and H3K27me3, along with decreased H3K9ac and H3K27ac, have been observed in the cerebral cortex and hippocampus of animal models exhibiting cognitive deficits (50). H3K27ac, associated with many immune-related genes, decreases over time, promoting inflammatory activation in the brain and exacerbating cognitive impairment (51). These changes in histone acetylation not only heighten stress responses but also activate immune inflammation, leading to upregulation of H3K4me2 and H3K27me2, increased expression of stress response proteins, and cognitive decline (52). Cognitive dysfunction correlates with increased H3K4me2 levels and heightened inflammation (52). Overall, histone methylation is implicated in the activation of stress and inflammatory pathways that underlie neurodegenerative processes. Luccarini et al. reported that oleuropein increased H3K9ac and acetylation at lysine 4 on histone 5 in the cortex and hippocampus of transgenic mice (50). Similarly, caloric restriction was shown to reduce H3K4me2 levels in aging cognitively impaired mice (52), while exifone treatment upregulated H3K9ac in human neuronal models of neurodegeneration (51). Our findings align with these observations, demonstrating that SCO treatment significantly increases H3K4me2 and H3K27me2 and decreases H3K9ac and H3K27ac levels, whereas hesperetin reverses these changes in mice with cognitive impairment. These findings suggest that the regulation of histone modifications may complement the activation of the TrkB/AKT/CREB/BDNF signaling pathway observed in the present study. Histone acetylation, particularly H3K9ac and H3K27ac, is associated with an open chromatin structure that facilitates the transcription of genes involved in synaptic plasticity and memory formation, including BDNF. Because CREB functions as a key transcription factor regulating BDNF expression, increased histone acetylation may enhance CREB-dependent transcriptional activity and thereby support long-term synaptic plasticity and memory consolidation. Conversely, repressive histone methylation, particularly H3K27 methylation, is associated with chromatin condensation and reduced neuronal gene expression (53). Therefore, the restoration of histone acetylation together with the reduction of repressive histone methylation by hesperetin may provide an epigenetic mechanism that cooperates with TrkB/AKT/CREB/BDNF signaling to promote neuronal survival, synaptic plasticity, and cognitive function.

HDACs play a critical role in learning and memory, and their dysregulation has been linked to cognitive decline in brain aging and neurodegenerative diseases (54). Among class I HDACs, HDAC2 and HDAC4 are particularly influential in cognitive processes (20). Notably, HDAC2-deficient mice exhibit increased synaptic density and enhanced memory performance (22). In our study, SCO administration led to decreased SIRT1 expression and elevated levels of HDAC2 and HDAC4. In contrast, hesperetin treatment significantly activated SIRT1 while suppressing HDAC2 and HDAC4 expression. These findings align with previous reports. For example, Liu et al. demonstrated that dexmedetomidine alleviates neuroinflammation via the HDAC2/HIF-1α pathway in mice with cognitive dysfunction (20). Similarly, Thomas et al. reported that resveratrol attenuates HDAC4 expression in streptozotocin-induced cognitive impairment (22). SIRT1, a NAD^+^-dependent class III HDAC (sirtuin), is essential for maintaining synaptic plasticity, learning, and memory and has been found to be reduced in the cerebral cortex of patients with AD (21). Furthermore, Sun et al. reported that curcumin activates SIRT1 in neurotoxic rat cortical neurons (55), while Xu et al. demonstrated that bisdemethoxycurcumin upregulates SIRT1 to mitigate oxidative stress in a mouse model of neurodegenerative disease (21). Our findings suggest that hesperetin ameliorates SCO-induced cognitive impairment by reducing neuronal damage and improving memory function. These effects are mediated through modulation of apoptosis and inflammation, activation of the TrkB/CREB/BDNF signaling pathway, and regulation of histone modifications.

## Conclusions

This study demonstrates that hesperetin administration in SCO-treated C57BL/6 mice mitigates brain tissue damage, including neuronal shrinkage, and improves memory performance. Hesperetin also effectively suppressed the expression of inflammatory cytokines and *NF-*κ*B* target genes associated with cognitive impairment. These neuroprotective effects are mediated by activation of the TrkB/CREB/BDNF signaling pathway, which alleviates memory impairment and promotes beneficial histone modifications. Collectively, the findings support the potential of hesperetin as a safe and effective compound for reducing oxidative stress-induced neuronal damage and for preventing neurodegenerative disorders. Therefore, hesperetin emerges as a promising bioactive therapeutic agent for counteracting synaptic loss and treating cognitive impairment.
